# Stroke and frailty index: a two-sample Mendelian randomisation study

**DOI:** 10.1007/s40520-024-02777-9

**Published:** 2024-05-22

**Authors:** Jiangnan Wei, Jiaxian Wang, Jiayin Chen, Kezhou Yang, Ning Liu

**Affiliations:** 1 Department of Nursing, Zhuhai Campus of Zunyi Medical University, No. 368 Jinwan Road, Zhuhai, Guangdong China; 2 Department of Fundamentals, Department of Basic Teaching and Research in General Medicine, Zunyi Medical University Zhuhai Campus, Zhuhai, Guangdong China

**Keywords:** Stroke, Frailty, Mendelian randomization, Genetic analyses

## Abstract

**Introduction:**

Previous observational studies have found an increased risk of frailty in patients with stroke. However, evidence of a causal relationship between stroke and frailty is scarce. The aim of this study was to investigate the potential causal relationship between stroke and frailty index (FI).

**Methods:**

Pooled data on stroke and debility were obtained from genome-wide association studies (GWAS).The MEGASTROKE Consortium provided data on stroke (N = 40,585), ischemic stroke (IS,N = 34,217), large-vessel atherosclerotic stroke (LAS,N = 4373), and cardioembolic stroke (CES,N = 7 193).Summary statistics for the FI were obtained from the most recent GWAS meta-analysis of UK BioBank participants and Swedish TwinGene participants of European ancestry (N = 175,226).Two-sample Mendelian randomization (MR) analyses were performed by inverse variance weighting (IVW), weighted median, MR-Egger regression, Simple mode, and Weighted mode, and heterogeneity and horizontal multiplicity of results were assessed using Cochran’s Q test and MR-Egger regression intercept term test.

**Results:**

The results of the current MR study showed a significant correlation between stroke gene prediction and FI (odds ratio 1.104, 95% confidence interval 1.064 − 1.144, P < 0.001). In terms of stroke subtypes, IS (odds ratio 1.081, 95% confidence interval 1.044 − 1.120, P < 0.001) and LAS (odds ratio 1.037, 95% confidence interval 1.012 − 1.062, P = 0.005). There was no causal relationship between gene-predicted CES and FI. Horizontal multidimensionality was not found in the intercept test for MR Egger regression (P > 0.05), nor in the heterogeneity test (P > 0.05).

**Conclusions:**

This study provides evidence for a causal relationship between stroke and FI and offers new insights into the genetic study of FI.

**Supplementary Information:**

The online version contains supplementary material available at 10.1007/s40520-024-02777-9.

## Introduction

Stroke is a cerebrovascular lesion caused by sudden cerebrovascular injury with high morbidity, disability, mortality and recurrence rates [1]. Stroke is the second leading cause of death worldwide, with an annual death rate of about 5.5 million [2]. Studies have shown that the incidence of stroke increases dramatically with age, with about three-quarters of all strokes occurring in people over the age of 65 [3]. The United Nations Population Prospects predicts that the number of people aged 60 years and older will reach 2.1 billion globally by 2050 and 3.2 billion by 2100 [4]. Elderly stroke patients suffer from long-term sequelae such as incapacitation, emotional deficits, and cognitive disorders [5], and because of the long duration of illness, high medical costs, and poor adherence to treatment, elderly stroke patients impose a heavy burden on society, families, and patients [6].

Frailty, characterized by age-related multisystem dysfunction, is a major public health problem in older adults [7, 8]. Studies show that frailty is common in strokes, with at least a quarter of stroke victims being physically frail [9]. Understanding the potential association between frailty and senescence-­related diseases and the underlying mechanisms may facilitate the individualized management and early interventions of frail patients.FI reflects the accumulation of physiological deficits in various systems of the body, and assesses frailty by calculating the number of deficits in the health variables, taking into account the effects of physical, psychosocial, and social factors on the human body [10]. Studies have shown that stroke frailty can be assessed by physicians or researchers in the early stages of stroke by means of a scale survey, and studies have confirmed the applicability of the FI in stroke patients [11].

Randomized controlled trials (RCTs) are the gold standard of clinical evidence and are widely used to infer causality, mainly by eliminating confounding bias through randomized grouping. However, randomized controlled trials require a great deal of time, money, and human resources; therefore, RCT studies are difficult to perform in many medical studies. Mendelian randomization (MR) is a genetic epidemiology study design method that allows for the exploration of causal relationships between exposures and outcomes through the use of genetic variation as an instrumental variable (IV) [12]. Due to the ability to overcome the effects of potential confounding and reverse causation, MR methods have been increasingly used in observational studies in recent years [13]. MR research has been facilitated by the discovery in biology of a large number of genetic variants that are strongly associated with specific traits, and by the public release of hundreds of thousands of pooled data on the association of exposures and diseases with genetic variants from many large-sample genome-wide association studies (GWAS),which have allowed researchers to estimate genetic associations in large-sample data.The method of MR, with its use of genetic variables, allows for the avoidance of reverse causality and minimizes the interference of environmental factors in a manner similar to that of RCTs [14].

In this study, we aimed to investigate the causal relationship between stroke and frailty index using MR methods.

## Materials and methods

### Mendelian randomization assumptions

**Fig. 1:**
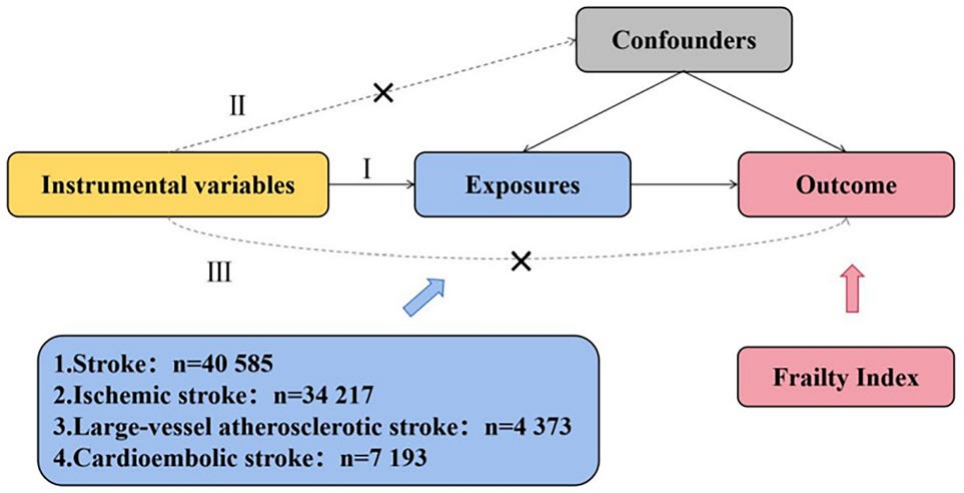
Design and main assumptions of our Mendelian randomization study

The MR method is an instrumental variables analysis that uses genetic variants as proxies for exposure. As in Fig. 1, the MR analysis relies on 3 important assumptions: (1) instrumental variables are closely related to exposure factors; (2) instrumental variables are independent of confounding factors; and (3) instrumental variables affect outcome only through exposure and not through other means [15].

### Data sources and SNP selection for stroke

The exposure factor for this study was defined as stroke, and data were obtained from the MEGASTROKE consortium, including 446,696 individuals of European ancestry (406,111 non-stroke cases and 40,585 stroke cases); the total number of ischemic stroke cases was 34,217, including 4373 large-vessel atherosclerotic stroke (LAS) cases, 5386 small-vessel stroke cases, and 7193 cardiac stroke cases.

### Data sources and SNP selection for frailty index

Frailty is commonly defined using the Frailty Index (FI) [16]. In this MR study, frailty was measured according to the FI, which was calculated based on the accumulation of 44–49 self-reported health deficits over the life course.Summary statistics for the FI were obtained from the most recent GWAS meta-analysis of UK BioBank participants and Swedish TwinGene participants of European ancestry (N = 175,226).

### Genetic instrumental variable selection

To meet the above hypotheses, the specific instrumental variables screening criteria were: (1) the association of single nucleotide polymorphism(SNPs) with single amino acids was genome-wide significant (P < 5 × 10^−8^); (2) the linkage disequilibrium (LD) between SNPs was calculated using the European population genome of 1000 individuals as the reference template, and SNPs with r^2^ < 0.001 and physical genetic distance > 10,000 kb were screened. (3) remove SNPs with minor allele frequencies < 0.01;(4) exclude SNPs with F values < 10 to avoid weak instrumental bias,and the following formula was used to calculate the F statistic [17]: F statistic = R^2^(N–2)/(1–R^2^). R^2^ = 2 × EAF × (1–EAF) × β^2^.The F values were all > 10, which indicated that our IVs were not biased by weak instrument. (5) apply MR Steiger [18] to assess the causal direction of each SNP on exposure and outcome, and exclude SNPs with reverse causality.

### Statistical analysis

#### Two-sample Mendelian randomization analysis

We performed two-sample MR analyses using the inverse-variance-weighted method as the primary approach.Four other MR methods based on different model assumptions were also used for the analysis: weighted median method [19], MR-Egger regression [20], Weighted mode and Simple mode. According to the MR-Egger regression method, intercepts different from the origin can be used to assess potential pleiotropic effects [21]. These various MR methods can test the stability and reliability of association under different assumptions.

### Sensitivity and power analysis

In this study, MR-Egger intercept was used to detect horizontal multiplicity, and if the intercept term in MR-Egger intercept analysis was statistically significant compared to 0, it indicated that the study had horizontal multiplicity [22]; Cochran Q test was applied to determine the heterogeneity of SNPs [23], and if the Cochran Q statistic test is statistically significant and proves that the analysis results have significant heterogeneity, then focus on the results of the random effects IVW method; using the Leave-one-out sensitivity test for sensitivity analysis [24], each SNP is eliminated in turn, and the remaining The MR results are robust if the remaining SNPs are not significantly different from the total results.The above methods were implemented using the TwoSampleMR package in the R 4. 2. 3 software with a test level of α= 0. 05.

## Results

### Instrumental variable

According to the screening criteria of the instrumental variables in this study, all strokes, ischemic strokes, atherosclerotic strokes of large arteries, and cerebral embolic strokes were finally screened for 17 SNP_stroke_, 18 SNP_IS_, 4 SNP_LAS_, and 4 SNP_CES_, respectively. Table 1 shows 18 SNPs significantly associated with ischemic stroke.The MR-Egger regression intercepts were b_stroke_ = 0.0179 (P = 0.8888), b_IS_ = 0.1234 (P = 0.2288), b_LAS_ = − 0.0121 (P = 0.8912), and b_CES_ = 0.0306 (P = 0.3773), respectively. That is, there was no genetic pleiotropy between the screened SNPs and the outcome frailty index, and thus the Mendelian randomization method was a valid method for causal inference in this study.

**Table 1:** Genetic variants significantly associated with ischemic stroke

SNP	Chr	Effect allele	Non-effect allele	Effect allele frequency	Effect size	Standard error	*p*-value
rs1052053	1	G	A	0.401	− 0.0576	0.0087	4.48E−11
rs1052054	19	G	A	0.6506	0.0479	0.0087	3.58E−08
rs1052055	5	G	A	0.1761	− 0.0719	0.0124	7.51E−09
rs1052056	16	A	G	0.3057	0.0609	0.0095	1.28E−10
rs1052057	1	A	G	0.405	0.0536	0.0088	1.34E−09
rs1052058	11	T	C	0.1281	0.08	0.0145	3.33E−08
rs1052059	7	A	G	0.2264	0.0759	0.0102	9.25E−14
rs1052060	12	C	T	0.5479	− 0.0751	0.0098	2.17E−14
rs1052061	12	T	C	0.3814	− 0.0495	0.0089	3.21E−08
rs1052062	7	T	C	0.2277	− 0.0656	0.0113	6.55E−09
rs1052063	15	A	G	0.333	0.0519	0.0094	2.88E−08
rs1052064	6	A	G	0.1372	0.0832	0.014	2.83E−09
rs1052065	4	C	T	0.3078	0.0564	0.0092	7.43E−10
rs1052066	4	T	A	0.3257	0.0784	0.0096	3.50E−16
rs1052067	12	C	A	0.407	− 0.0484	0.0089	4.93E−08
rs1052068	9	T	C	0.5355	0.0514	0.0084	1.05E−09
rs1052069	13	G	A	0.7614	0.0615	0.0101	9.19E−10
rs1052070	17	C	T	0.188	0.0893	0.0162	3.63E−08

*Chr* chromosome, *SNP* single nucleotide polymorphism

### Mendelian randomization analysis

**Fig. 2:**
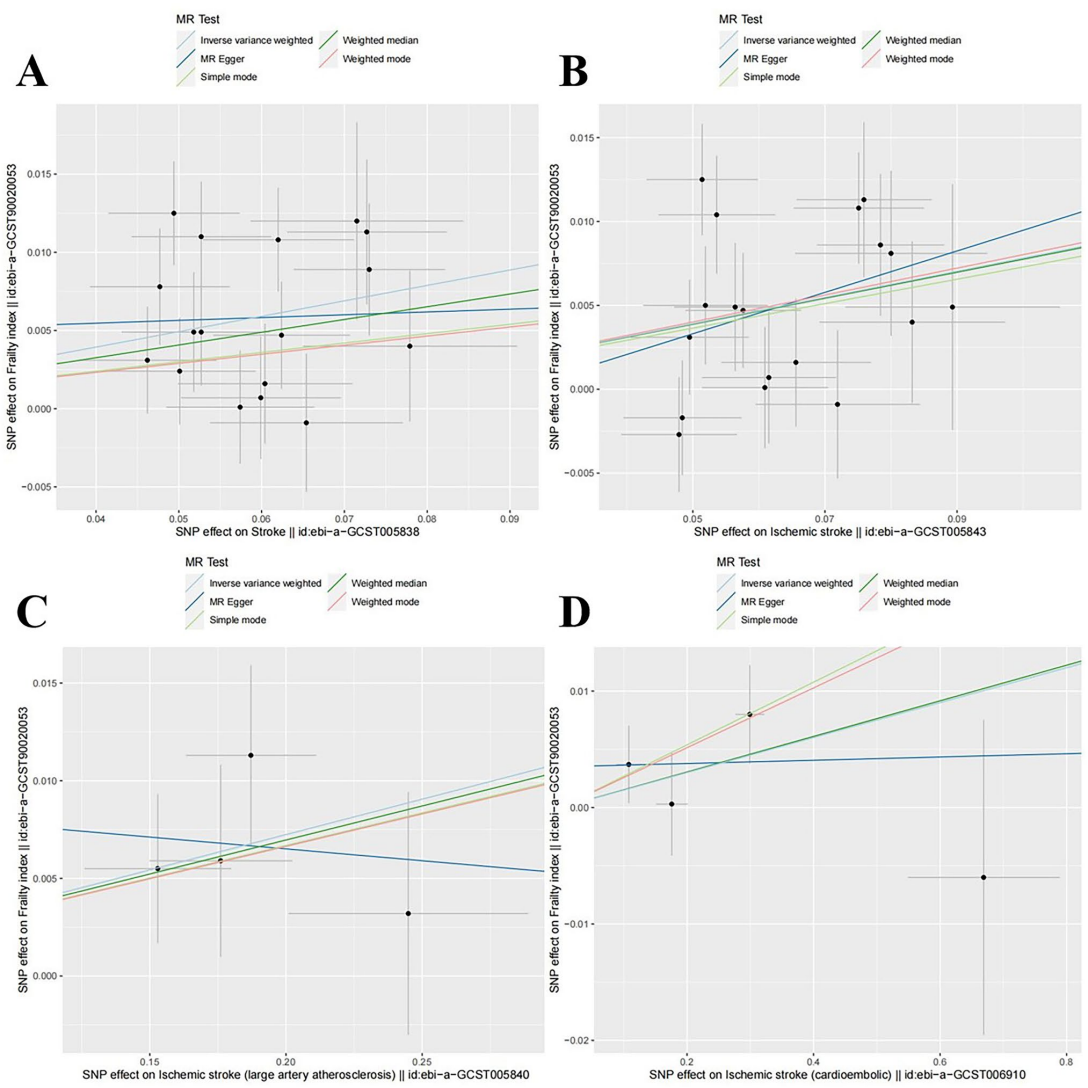
Scatter plots of the results of 5 MR methods: **A** Stroke; **B** IS; **C** LAS; and **D** CES

The results of the ivw method showed a significant relationship between stroke (OR = 1.104, 95% CI 1.064–1.144, p < 0.001), IS (OR = 1.081, 95% CI 1.044–1.120, p < 0.001), LAS (OR = 1.037, 95% CI 1.012–1.062, p = 0.005), and FI There was a causal relationship between. In Weighted median method analysis, stroke (OR = 1.085, 95% CI 1.037–1.135, p < 0.001), IS (OR = 1.081, 95% CI 1.034–1.129, p < 0.001), LAS (OR = 1.035, 95% CI 1.005–1.066, and p = 0.026) and FI were also causally related. However, there was no evidence to support a causal relationship between CES and FI. MR estimates and efficacy analyses for stroke and FI are shown in Table 2, and scatter plots of MR analyses for the 5 methods are shown in Fig. 2.

**Table 2:** Five methods of MR regression results for four types of stroke

Type	Method	b	SE	OR(95%CI)	*p*-value
Stroke	MR-Egger	0.018	0.126	1.018(0.795–1.303)	0.889
	Weighted median	0.082	0.023	1.085(1.037–1.135)	< 0.001
	IVW	0.099	0.019	1.104(1.064–1.144)	< 0.001
	Simple mode	0.060	0.043	1.072(0.975–1.156)	0.186
	Weighted mode	0.058	0.041	1.060(0.977–1.149)	0.179
IS	MR-Egger	0.123	0.099	1.131(0.932–1.373)	0.229
	Weighted median	0.078	0.023	1.081(1.034–1.129)	< 0.001
	IVW	0.078	0.018	1.081(1.044–1.120)	< 0.001
	Simple mode	0.073	0.045	1.076(0.986–1.174)	0.121
	Weighted mode	0.080	0.047	1.084(0.988–1.188)	0.106
LAS	MR-Egger	− 0.012	0.078	0.988 (0.835–1.141)	0.891
	Weighted median	0.035	0.016	1.035 (1.005–1.066)	0.026
	IVW	0.036	0.013	1.037(1.012–1.062)	0.005
	Simple mode	0.033	0.021	1.034 (0.992–1.076)	0.217
	Weighted mode	0.033	0.023	1.034 (0.990–1.078)	0.238
CES	MR-Egger	0.001	0.020	1.001(0.961–1.042)	0.952
	Weighted median	0.015	0.012	1.015 (0.992–1.039)	0.194
	IVW	0.015	0.010	1.015 (0.996–1.035)	0.130
	Simple mode	0.027	0.019	1.027 (0.990–1.064)	0.247
	Weighted mode	0.026	0.014	1.026 (0.999–1.053)	0.156

**Fig. 3:**
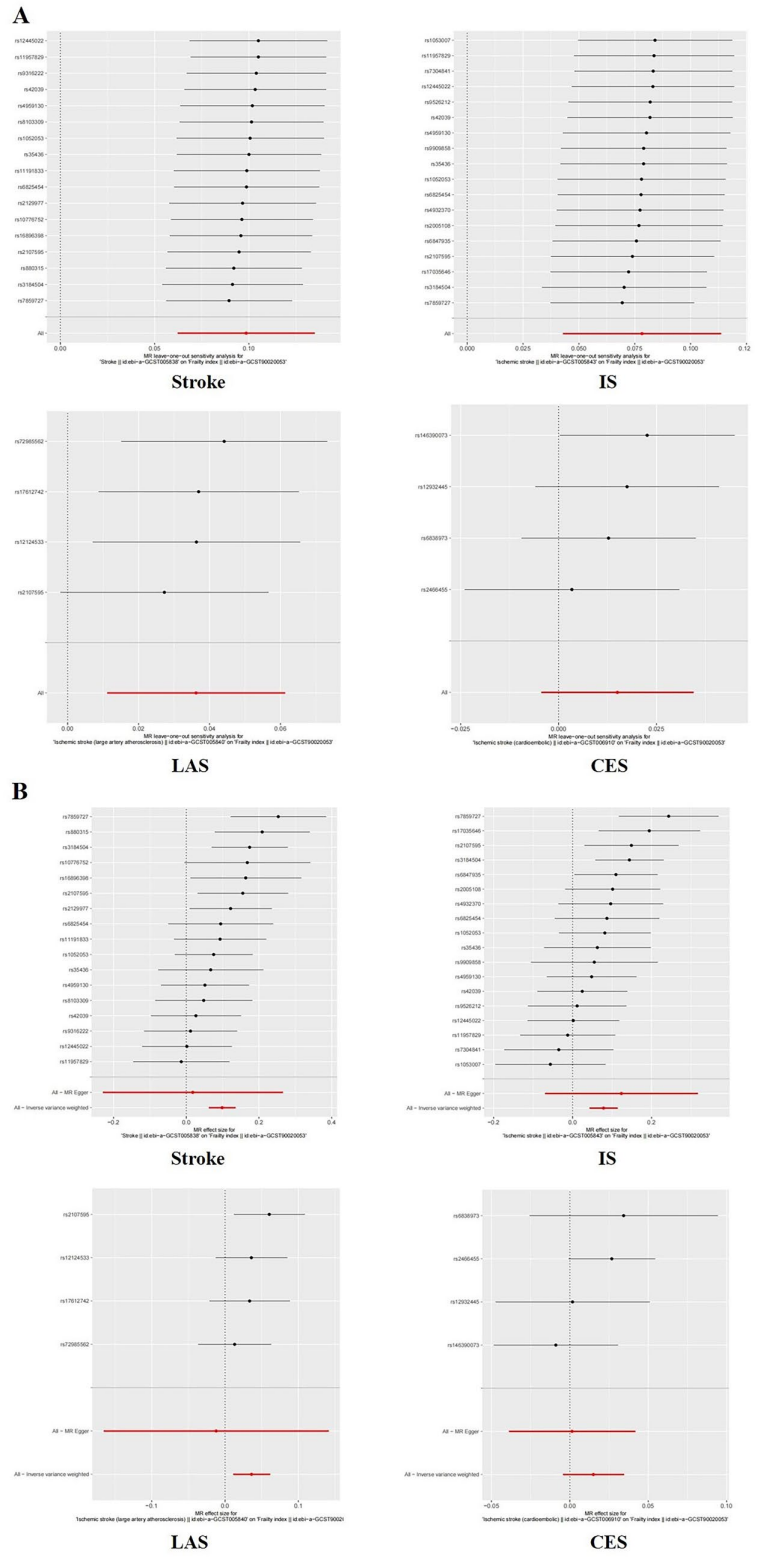
Sensitivity analysis of the association between genetically predicted Stroke and FI. **A** “Leave-one-out” sensitivity analysis results; **B** Forest plot.IS,ischaemic stroke;LAS,large artery atherosclerosis stroke;CES, cardioembolism stroke

In this study Cochran’s Q test was used to assess the heterogeneity of the results which was done by both IVW and MR-Egger’s analyses.The results of CochranQ test for IVW method showed Q_stroke_ = 22.193, Q_IS_ = 26.370, Q_LAS_ = 1.813, Q_CES_ = 1.208.MR-Egger’s CochranQ test showed Q_stroke_ = 21.590, Q_IS_ = 26.016, Q_LAS_ = 1.421, Q_CES_ = 1.084.Regarding the results of the horizontal multivariate test, it was assessed using MR Egger’s intercept term. The p-value of heterogeneity and horizontal polytropy was greater than 0.05, so there was no heterogeneity and horizontal polytropy in this study, so the results had a weak risk of bias and high reliability. In addition, we performed Leave-one-out analysis, and the results showed that after removing each SNP in turn, the b-values of the remaining SNPs were (0.089–0.105), (0.694–0.841), (0.012–0.150), and (0.012–0.027), respectively, with p < 0.01. The b-values were all > 0, and the directions were all the same, indicating that removing either SNP had little effect on the results, and a positive causal association between stroke and frailty index was still observed. Sensitivity analyses and forest plots of the association between gene-predicted stroke and FI are shown in Fig. 3.

## Discussion

In this study, based on large-scale GWAS pooled data, a two-sample MR method was used to analyze the causal association between stroke, IS, LAS, CES and FI. The results showed that stroke, IS, LAS, and CES were risk factors for FI. Further sensitivity analyses showed the consistency and reliability of these results.

Our finding that stroke may cause frailty provides evidence for early observational studies.Previous studies have reported an association between stroke and frailty [25]. The research results show that 12.8% of ischemic stroke patients and 10.3% of hemorrhagic stroke patients are already in a weakened state before stroke, and the degree of weakness worsens after stroke [26]. Stroke may accelerate the occurrence and development of physical weakness. In a large-scale assessment of debility, Hanlon et al. found that debility was common among stroke survivors. Rodriguez et al. showed an overall prevalence of frailty of 15.2% in older adults and a positive correlation between frailty and stroke in a survey of eight urban and four rural areas in eight countries, including Cuba, the Dominican Republic, Puerto Rico, Venezuela, Peru, Mexico, China, and India.Palmer et al. [27] found that the incidence of frailty was twice as high in stroke patients as in those who did not have a stroke [28]. Rowan et al. found frailty in about a quarter of acute stroke patients through a cross-sectional survey [11]. Stroke increases the risk of debilitation, and the prevalence of debilitation varies widely by region between countries, while debilitation imposes a serious burden on stroke patients and reduces their quality of life.

Exploring the causal relationship between these two comorbidities may be difficult because they share the same risk factors, such as hyperglycemia and Dyslipidemia. In addition,Evans et al showed that neurological deficits after stroke may exacerbate the phenotypic features of frailty, that hemodynamic changes in central and peripheral vasculature occur with age, and that frailty is associated with impaired brain self-regulation. And that a history of previous stroke is an important factor in the transition from robust to debilitated patients and in the worsening of the debilitating trajectory [29]. Hanotier et al. pointed out that prolonged malnutrition can easily lead to electrolyte disorders in the elderly. Once a stroke occurs, due to insufficient nutrition intake, electrolyte disorders worsen, and body mass sharply decreases, ultimately leading to frailty in the elderly [30]. Stroke patients have varying degrees of neurological dysfunction, which can affect the number of skeletal muscles to a certain extent [31], leading to the occurrence of sarcopenia, thereby reducing limb muscle strength and grip strength, and increasing the risk of physical weakness.

Zhu et al. [8] conducted a two-way Mendelian randomisation study of FI and stroke and found that FI was significantly associated with stroke in both directions of the outcome, but stroke-related subgroup analyses were not performed, which somewhat supports our findings. Liu et al. [32] found an implied association between FI and any stroke, and FI was associated with a high risk of LAS, but there was no causal association between FI and IS and small-artery stroke. There was no causal relationship.However, our study found that stroke, IS, and LAS were all causally related to FI, which may be due to the fact that our study was conducted in a different direction than theirs.

The main strengths of this study include the use of MR for causal inference and analyzing a large sample group. MR methods can effectively avoid the drawbacks of uncertain residual confounding and reverse causality that exist in traditional observational study methods [21]. Data on stroke and frailty are derived from existing large GWAS, which allows for more precise assessment of effect sizes than individual-level data or results from studies with limited sample sizes. Inevitably, there are some limitations. First, the two-sample Mendelian randomization method assumes a correlation between the exposure factor and the outcome, and the MR method is not applicable if the relationship is nonlinear. Second, the results of the analysis in this study are based only on populations of European origin, so further research and validation are needed for generalization to other populations. Finally, database statistics are difficult to analyze stratified by sex or age, which may lead to biased findings.

## Conclusions

In summary, we found a causal relationship between stroke and its subtypes and debility by two-sample MR analysis. Further studies are needed to elucidate the potential mechanisms underlying the various causal relationships between stroke subtypes and debility.

### Supplementary Information

Below is the link to the electronic supplementary material.

Supplementary file 1 (DOCX 432 KB)

## Data Availability

All data are publicly available. Detailed information for these datasets is summarized in supplementary material.
