# Supplementary material for: Stroke and frailty index: a two-sample Mendelian randomisation study
Source: Aging Clin Exp Res. 2024 May 22;36(1):114. doi: 10.1007/s40520-024-02777-9 (PMC11111486; doi:10.1007/s40520-024-02777-9)
Supplement: Supplementary file 1 — (DOCX 432 KB) [file 40520_2024_2777_MOESM1_ESM.docx]

**Supplementary Table S1. Data sources of the instrumental variables**

| **Trait** | **Sample Size** | **Cases** | **Controls** | **Number of SNPs** | **Populations** | **Dataset** | **PMID** | **Year** |
| --- | --- | --- | --- | --- | --- | --- | --- | --- |
| Stroke | 446,696 | 40,585 | 406,111 | 7,633,440 | European | ebi-a-GCST005838 | 29531354 | 2018 |
| IS | 440,328 | 34,217 | 406,111 | 7,537,579 | European | ebi-a-GCST005843 | 29531354 | 2018 |
| LAS | 150,765 | 4,373 | 406,111 | 7,992,739 | European | ebi-a-GCST005840 | 29531354 | 2018 |
| CES | 211,763 | 7,193 | 406,111 | 8,271,294 | European | ebi-a-GCST006910 | 29531354 | 2018 |
| FI | 175,226 | - | - | 7,589,717 | European | ebi-a-GCST90020053 | 34431594 | 2021 |


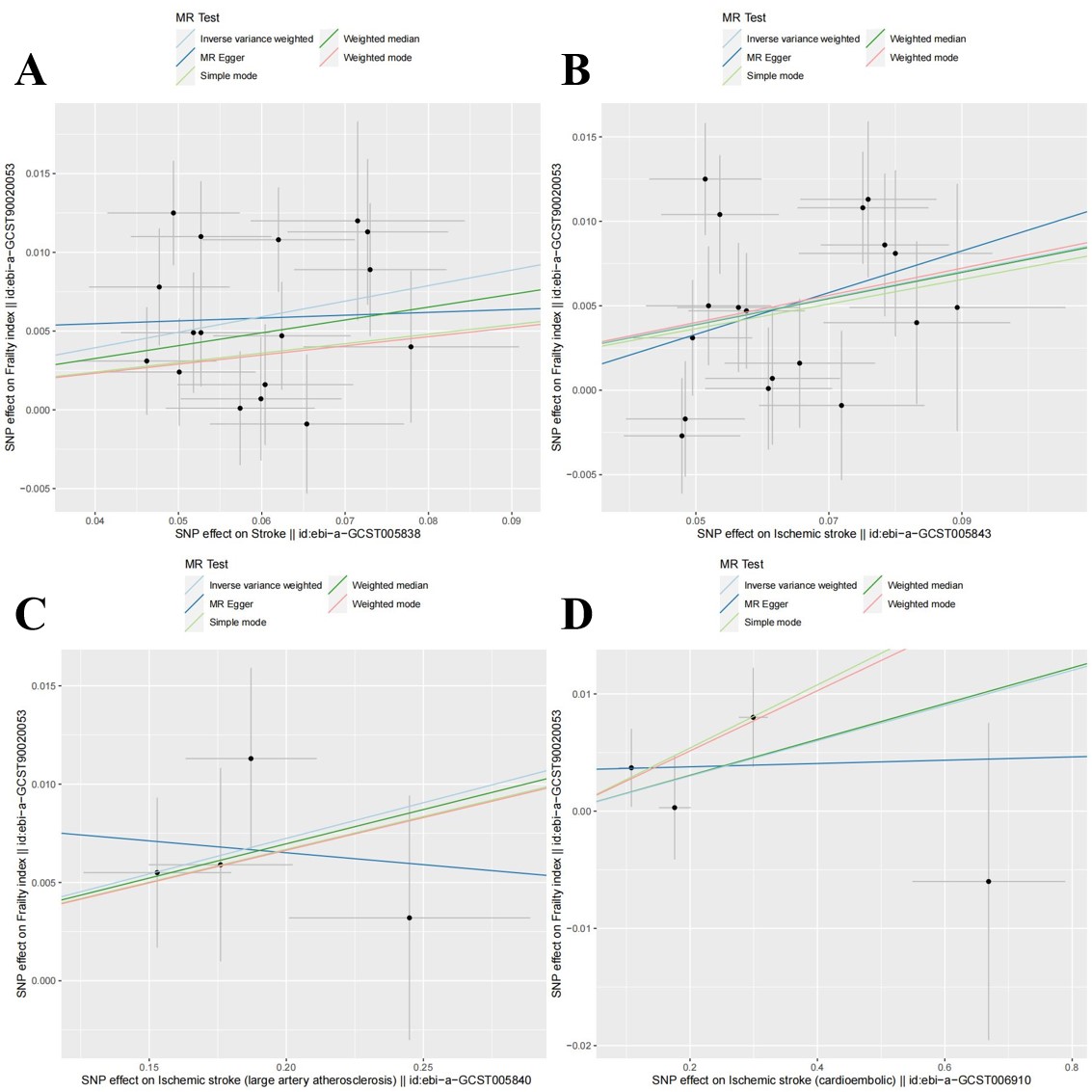


**Fig. 2** Scatter plots of the results of 5 MR methods: (A) Stroke; (B) IS; (C) LAS; and (D) CES.

**
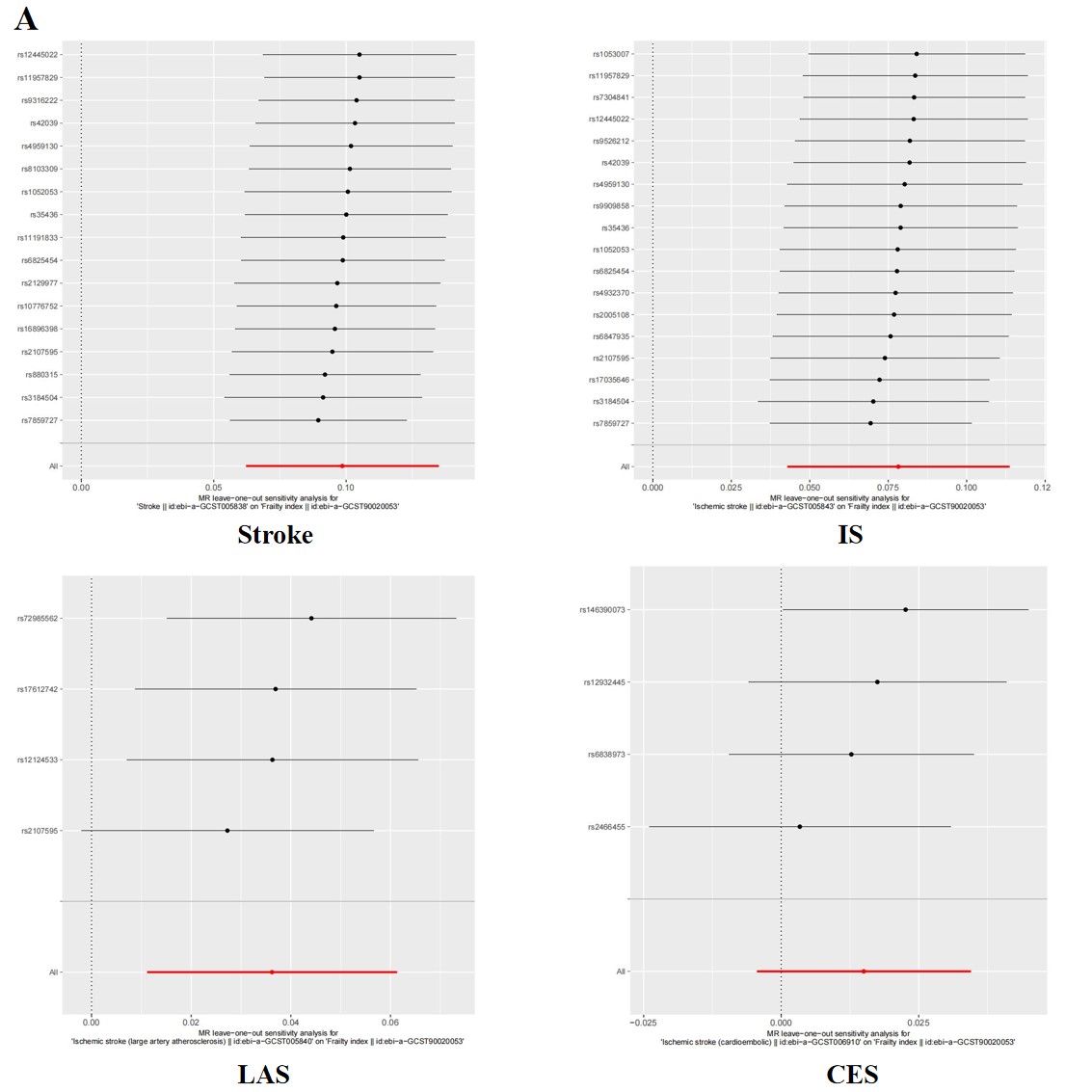

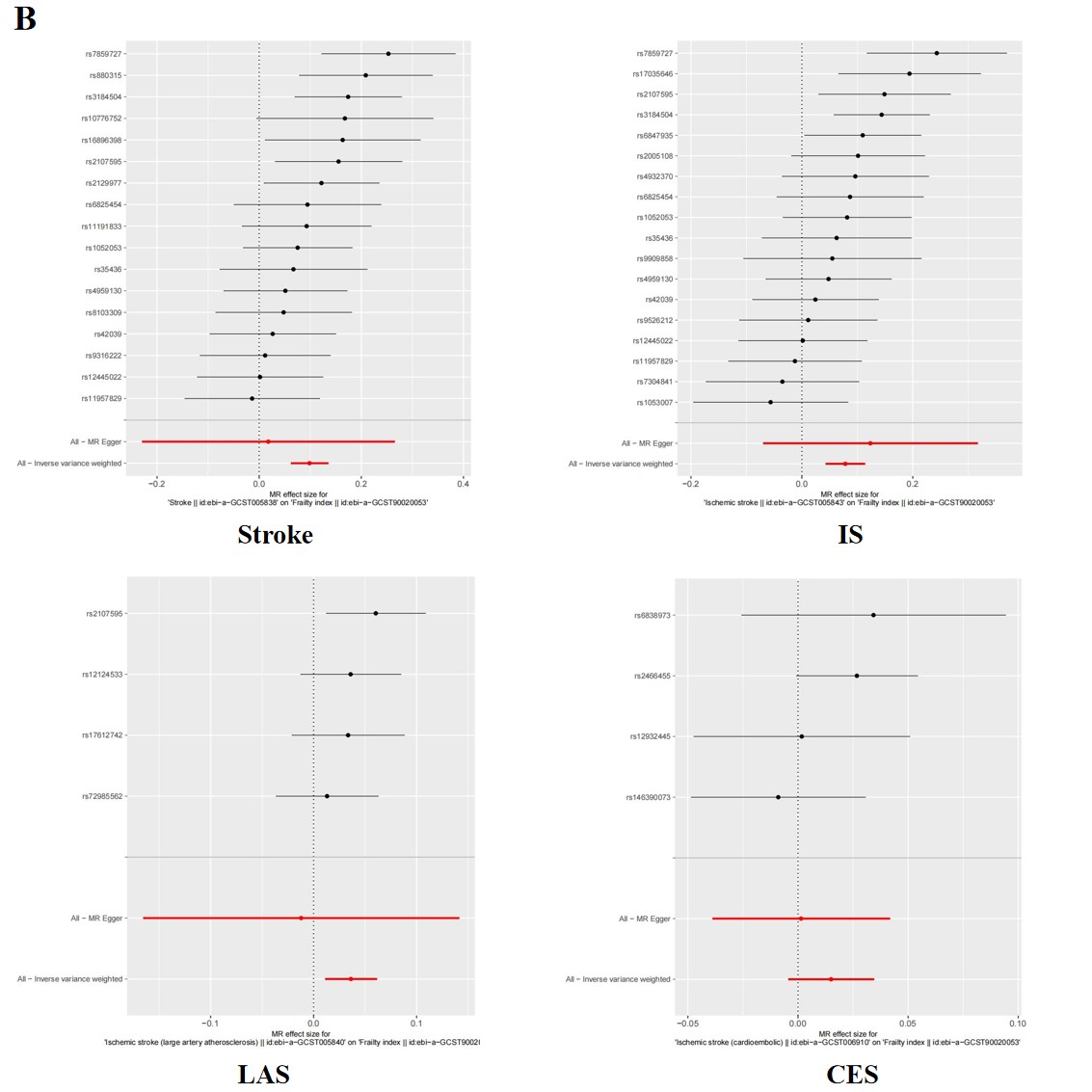
**

**Fig. 3** Sensitivity analysis of the association between genetically predicted Stroke and FI.(A) “Leave-one-out” sensitivity analysis results; (B) Forest plot.IS,ischaemic stroke;LAS,large artery atherosclerosis stroke;CES, cardioembolism stroke.
